# Synonym set extraction from the biomedical literature by lexical pattern discovery

**DOI:** 10.1186/1471-2105-9-159

**Published:** 2008-03-24

**Authors:** John McCrae, Nigel Collier

**Affiliations:** 1 National Institute of Informatics, Hitotsubashi 2-1-2, Chiyoda-ku, Tokyo, 101-8430, Japan

## Abstract

**Background:**

Although there are a large number of thesauri for the biomedical domain many of them lack coverage in terms and their variant forms. Automatic thesaurus construction based on patterns was first suggested by Hearst [1], but it is still not clear how to automatically construct such patterns for different semantic relations and domains. In particular it is not certain which patterns are useful for capturing synonymy. The assumption of extant resources such as parsers is also a limiting factor for many languages, so it is desirable to find patterns that do not use syntactical analysis. Finally to give a more consistent and applicable result it is desirable to use these patterns to form synonym sets in a sound way.

**Results:**

We present a method that automatically generates regular expression patterns by expanding seed patterns in a heuristic search and then develops a feature vector based on the occurrence of term pairs in each developed pattern. This allows for a binary classifications of term pairs as synonymous or non-synonymous. We then model this result as a probability graph to find synonym sets, which is equivalent to the well-studied problem of finding an optimal set cover. We achieved 73.2% precision and 29.7% recall by our method, out-performing hand-made resources such as MeSH and Wikipedia.

**Conclusion:**

We conclude that automatic methods can play a practical role in developing new thesauri or expanding on existing ones, and this can be done with only a small amount of training data and no need for resources such as parsers. We also concluded that the accuracy can be improved by grouping into synonym sets.

## Background

Synonymy is one of the most important relations found between different terminology and is of critical importance for building high quality text mining systems for biomedical literature. Thesauri which list synonymous terms have been found to be useful for improving the results of information retrieval systems [2] and synonymy relations are encoded in many ontologies, for example Gene Ontology [3], and biological databases such as SWISS-PROT [4]. General thesauri, such as WordNet, give relatively poor coverage of specialised domains and thesauri often do not exist for many languages and domains. Domain specific thesauri and ontologies are expensive to construct, due to the scarcity of human expert resources, and often do not give sufficient variants of terminology. Against this background, automatic discovery of synonymy relations between terms has been shown to be useful for both maintaining and expanding existing ontologies [5] and for constructing ontologies [6], however the accuracy of such methods remains a major issue.

There has been a large amount of interest in constructing thesauri and ontologies automatically, not only for synonymy relations, which we study here, but also for hypernymy (general/specific) relations. Several methods have been suggested, for example, in Morin & Jacquemin [7] they explore using term variation (for example "mouth cancer" vs. "cancer of the mouth"), to detect synonyms, but many synonymous terms are not simple variants, so this method is limited. Distributional similarity, that is identifying terms by other terms which occur in close proximity, has also been shown to be effective for identifying synonymy, most notably the Latent Semantic Analysis method of Cederberg & Widdows [8]. As shown in Dumais *et al *[9], this can be used to improve the recall of a synonym classifier although at the cost of its precision. This method however lacks the ability to differentiate between specific semantic relations (for example synonymy, hypernymy, agent/disease).

Hearst [1] used patterns including "X such as Y" to detect a hypernymy relation between terms, however she chose these patterns by hand. Due to issues of accuracy and scalability to other relations, some work has gone into constructing these patterns automatically, notably in Snow *et al *[10]. Their pattern extractor was based on using a dependency grammar and so requires a grammar and a parser, which will limit applicability to those few languages where large coverage parsers have been developed. They then generated a large number of patterns and classified them by a logistic regression-based method. Finally they attempted to improve their results by using distributional similarity [11]. They also used a number of hand-chosen synonymy patterns to detect potential synonyms and used this to improve their detection rate. An attempt to find gene and protein name synonyms was explored in Yu *et al *[12], again they manually chose their patterns.

One of the disadvantages of most of these approaches is that they give only binary classification rather than outputting synonym sets. For practical applications of this problem a simple list of synonymous terms is much more desirable for several reasons, firstly the results are much simpler and easier to store and work with, as you need only list the groups instead of the synonymy relation between each pair. Also the result is self-consistent, so we will not get a result that X is synonymous to Y, which is synonymous to Z, but not that X is synonymous to Z. This may cause problems in applications where only a single term is use to represent the synset, for example a method based on usage of an ontology may require one main term for the synset. An attempt at improving the result of a term similarity classifier by graph clustering was explored in Ibekwe-Sanjuan & Sanjuan [13], however their method was not based on probabilities and the clustering method suggested would lead to questionable behaviours such as grouping a synset when the vast majority of links are not indicative of synonymy, hence the classifier must have a very high precision. Our work is most closely related to the work in Snow *et al *[10] for automatically discovering hypernyms. In order to find these patterns we chose to develop a system based on Soderland's WHISK system [14] as it is uses regular expression matching and so requires no prior knowledge of the syntax of the language. There they started with a base pattern consisting only of "*"s and slots and grew the pattern by replacing each of these "*"s with a word from their corpus. They then attempt to find a set of patterns which maximises the overall performance. We also wish to investigate this method in specific domains, and see if it is still feasible with a smaller seed set as the seed set in Snow *et al *[10] was the entirety of WordNet. For our experiment we chose to focus on the disease control domain, but our method could easily be applied to many domains in bio-medicine or other areas. We used the number of matches of each pattern for each term pair, to create a feature vector which could be used to statistically classify each term pair as synonymous or not. Finally we used probabilistic analysis to find the most likely set of synsets, based on modelling the output of the classifier as a probability graph.

### Data Collection

For our experiment we decided to use an existing ontology to provide a training set from which our system could develop patterns and then train a classifier. Our training set was drawn from the English section of the BioCaster ontology [15]. This ontology was put together by life scientists and its terms relate to some of the most important infectious diseases currently being tracked by epidemiologists. BioCaster was developed for the search and analysis of Internet news and research literature to enable the timely detection of possible disease outbreaks. As such it contains a range of lay terms and technical terms, and we choose 4 categories of terminology from the ontology: infectious diseases, symptoms (of infectious diseases), agents (e.g. viruses, bacteria) and hosts (e.g. mammals, birds). This was useful as it gave us an accurate basis of terminology relating to a specific biomedical domain.

To develop a test set we wished to find terms that occurred naturally and so our evaluation would represent the effectiveness of the method in actual implementations. We obtained the top 150 PubMed abstracts containing the term "infectious diseases", and manually extracted all the terms in the same four categories. We then grouped these words into synsets attempting to follow the same guidelines as used by the BioCaster ontology. Synonymy can be quite difficult to decide, with granularity being a major problem, in fact even established thesauri can differ in their definitions of synonymy as investigated by Burgun & Bodenreider [16]. We found the following problems frequently occurred.

1. **Modifiers: **Terms often appear with modifiers, such as "*acute *headache," and although many ontologies would list this as a property of the disease or symptom we decided to list terms separately. It is important to be careful not to confuse this with modifiers which do not change the meaning of the term, for example "*mental *retardation." More information on the effect of modifiers can be found in Bodenreider *et al *[17].

2. **Granularity: **Many terms may turn out to be used interchangeably as the difference in meaning is rarely important. An example of this is "HIV-1," which as the most common strain of HIV is often simply referred to as "HIV," however as these subtle differences in meaning may prove to be important we decided to separate such terms

3. **Domain-specific:**

(a) **Method of Transmission: **We decided to list terms differing in method of transmission separately, for example "*hospital-acquired *MRSA."

(b) **Point of Infection: **Terms that differ in only the point of infection were decided not to be synonymous, for example "syphilis" and "neurosyphilis."

(c) **Progression of Disease: **It was decided that terms used to describe a disease at a more advanced stage were not synonymous with the disease, for example "dengue fever" may develop into the more serious "dengue shock syndrome."

In total the BioCaster database consisted of 450 terms, grouped into 244 synsets, and containing 477 synonym pairs, similarly the test set consisted of 301 terms, 221 synsets and 101 synonym pairs, of which it shared 35 terms and 16 synonym pairs with the BioCaster database. We also had a second annotator group our 301 test terms into synonym groups and this list showed *κ *= 68.6% Cohen's agreement with our list, which illustrates that this problem is complicated by very subtle differences in meaning between our terms. We then extracted a large corpus from which we can build patterns and statistics. We simply did this by querying every term in both our training and test sets in the PubMed database. We chose the top 250 abstracts for each term ranked according to Entrez search's ranking, discarding any duplicate documents. This gave us a corpus consisting of 83,492 documents, and 1,506,042 sentences of which term pairs in our training set were found in 46,216 sentences.

## Method

We decided the best solution would be to first generate a large number of patterns using the training set. For each possible term pair we generate a feature vector which represents the frequency of matching that term pair using each of the patterns generated. This feature vector is then classified, and the posterior probability used in our synset formation algorithm described below.

It should be noted that we used the number of term pairs to calculate these scores, which gives a lower value than might be expected. For example if 4 terms were correctly identified out of a synset of 8 terms, then only 6 out of 28 synonym pairs have been identified, so the score is only 21.4% even though 50% of the terminology was recognised.

### Pattern Generation

**Figure 1:**
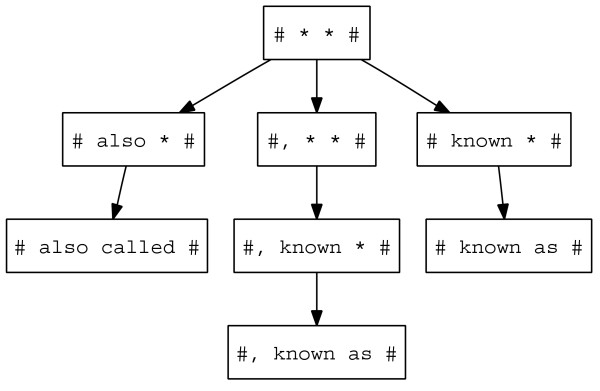
**Example Pattern Search**. A tree illustrating a exemplary search space for the pattern generation algorithm. The top of the tree is a seed pattern and the search continues by replacing one of the wild cards with term and punctuation to generate a set of patterns.

Our method for pattern generation is as follows we start with base patterns which consist of three basic operators *, #, and *(space)*, where * represents a sequence of word characters, *(space) *represents any white-space/punctuation between words, and # represents a term (matched case-invariantly). We generate new patterns by expanding a current pattern in the following ways: * may be replaced by any sequence of alphanumeric characters and *(space) *may be replaced by any sequence of non-word characters. By starting with a set of seed patterns consisting of only #s, *s and *(space*)s these patterns can be expanded to give us a search as in Figure 1. To expand a pattern we search the corpus by taking each synonym pair and replacing the #s with the terms. By considering each possible single expansion of a * or *(space) *our problem can be viewed as a tree search. Obviously this search space is huge and so we search it heuristically by best-first search. The algorithm is as follows:

### Pattern Generation Algorithm

**Input:**A set of base patterns *P*, a set of training term pairs $S=\{(t_{1},t_{2}),...(t_{n},{t^{\prime }}_{n})\}$, a corpus *C*.

**Output:**A set of patterns *P *sorted by a scoring metric

1. Add all the base patterns with a heuristic score to a heap *H*.

2. For a fixed number of iterations

(a) Select a pattern, *p*, with maximal score in *H*.

(b) Find all matches of this pattern using all term pairs in *S *in the corpus *C*

(c) For each * or *(space) *in *p*, find all matching strings in the corpus

(d) For each match to each * or *(space)*, add a new pattern to *H*

3. Output patterns *H *sorted by score

We experimented with a number of heuristics scoring metrics, including the number of pattern matches, however we found that this gave too strong a bias for terms which occurred very frequently, in particular we noted that many of the patterns contained terms from our training set, which is quite undesirable.

$$h_{1}=\frac{\#sentences\;matching\;pattern}{\#sentences\;in\;corpus}$$

We also report a pseudo-F-Measure given as below, where we judged a pattern match to be correct if it corresponded to a term in our training set. Note that as we can only consider a pattern match to be correct if it contains terms in our training set, the precision is under-estimated so this does not represent the true F-Measure associated with this pattern.

$$\begin{array}{c} h_{2}=\frac{(1+\alpha )\hat{p}\hat{r}}{\alpha \hat{p}+\hat{r}}\\ \hat{p}=\frac{\#correct\;pattern\;matches}{\#all\;pattern\;matches}\\ \hat{r}=\frac{\#synonym\;pairs\;found}{\#synonym\;pairs} \end{array}$$

Although this gave a good result it significantly increased the search time. In the end we decided that the best answer would be to use the number of synonym pairs found as this was readily computed and did not bias towards more common terminology. In many pattern generation methods, including the WHISK system, significant effort is made to develop a strong set of patterns, we designed this algorithm just to produce lots of reasonable results. The rationale behind this was that we wanted to give the synonym classifier the most information we could, and that too much complexity in the pattern generation duplicated the effort of the statistical classifier. The heuristic we used is as follows.

$$h_{3}=\frac{\#synonym\;pairs\;found}{\#synonym\;pairs}$$

We let the algorithm run for a fixed number of iterations, we chose this number to find approximately 10 times the number of patterns we plan to use in our classification.

### Synonymy Classification

To generate the feature vectors we found that many of the patterns were inflexible and matched very rarely, to combat this we simply allowed * and *(space) *to match *ε*, the empty string, by which we mean that allow a match with any of the *s or *(space)*s omitted. This allowed patterns such as # (* #) to match not only "*term *(or *abbrev*)," but also "*term *(*abbrev*)." We found this greatly improved the recall and the precision of the result.

We would expect some of the patterns generated to produce reasonably good precision and some relatively good recall, however by combining these all together we should be able to get a much better overall result. For this reason we view the problem as a statistical classification problem.

Once we have generated a number of patterns we simple use these to generate a feature vector for each synonym pair, this is simply given by the number of matches of pattern with the #s matching the terms in the candidate synonym pair. Although this in theory would require $\frac{n}{2}$(*n *- 1) feature vectors for *n *terms, in fact most pairs of terms do not occur close to each other at all in the corpus so they can be dropped (and their probabilities gained by classifying the zero vector). For our experiment we used the top 6000 patterns from the pattern generation algorithm. This then becomes a standard statistical classification problem, to which any statistical classification algorithm can be applied. We experimented with naïve Bayes, logistic regression, C4.5 and support vector machines with a number of kernel choices. We found that only logistic regression gave a useful spread of probabilities that could be used for synset formation so we built the synsets first with these probabilities and then we used logistic regression on the output of the strongest classifier (SVM).

### Synset formation

**Figure 2:**
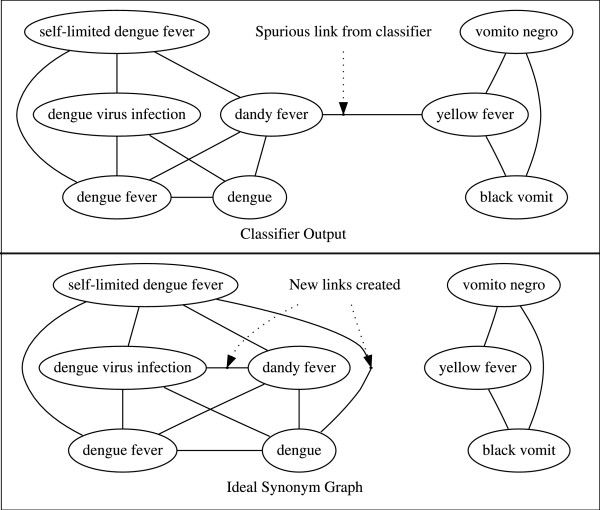
**Random Graph to Synsets**. Illustration of conversion from a random graph to a set of synsets. The top graph illustrates the random output from the classifier, which includes a spurious link from "dandy fever" to "yellow fever". This graph is corrected by forming into synsets removing the spurious link and adding two links which were not found by the classifier.

The results we gained from the statistical classification procedure gave only the probability of a particular term pair being synonymous. However we would expect every pair of terms in a synset to be synonymous and these binary classification results do not guarantee that such a transitivity relation exists. As such we shall assume that every pair of terms in a synset are synonymous and no pairs of terms in different synsets are synonymous (although this is technically incorrect as some words may be polysemous). This clearly leads to the result that synsets are complete graphs, so we can consider our goal as that of finding the closest set of complete sub-graphs to our random graph. As an example consider Figure 2, which shows a graph representation of the output on top, where the nodes represents terms, and they are connected if the classifier predicts the terms are synonymous. It is clear that the graph above should give two synsets as shown in the bottom graph.

Let {1..*N*} correspond to our terms, then our goal is to find a set $I^{*}=\{I_{1}^{*},...I_{n}^{*}\}$ where I* *exactly covers *{1..*N*} and I* maximises *c*(I*) given by

$$\begin{array}{c} c(\{I_{1},...,I_{K}\})=\sum_{k=1..K}c(I_{K})\\ c(I_{k})=\sum_{i\in I_{k}}\sum_{j\in I_{k}}\left(\log(P_{ij}\right)+\sum_{j\notin I_{k}}\log(1-P_{ij})) \end{array}$$

where *P*_*ij *_is the probability of the terms corresponding to i and j being synonymous as given by some statistical classification method (and *P*_*ii *_= 1). Note this logarithmic form is used as it is easier for further calculations and many of the output probabilities from our classifier were near 0 or 1. We also define the *inter-node cost*, *c*_*ij *_as

*c*_*ij *_= *log*(*P*_*ij*_) + *log*(*P*_*ji*_) - *log*(1 - *P*_*ij*_) - *log*(1 - *P*_*ji*_)

As the number of potential synsets is 2^*N*^, we can greatly reduce this problem by finding a small set of potential synsets *I *such that I* ⊂ *I *⊂ *P*({1..*N*}). Fortunately the majority of the output probabilities *P*_*ij *_are very small, so we can hope to have significant success by generating the set *I *by a branch and bound algorithm. We find a condition when a set and all of its super-sets are not optimal by observing that *J *∉ I* if *c*(*J *∪ {*k*}) <*c*(*J*) + *c*({*k*}) for some *k *∈ *I*.

**Lemma 1**: Let *J *⊂ {1..*N*} and *k *∉ *J *and *V *⊂ {1..*N*} such that *J *∩ *V *= ∅ and *k *∉ *V *. Then there is no set *K *such that *J *∪ {*k*} ⊂ *K *⊂ *J *∪ {*k*} ∪ *V*, *K *∉ I* if

1. ∑_*i*∈*J *_*c*_*ik *_< 0

2. -∑_*i*∈*J *_*c*_*ik *_> *max*_*V'*⊂*V*_∑_*i*∈*V' *_*c*_*ik*_

**Proof**: Follows directly from the inequality *c*(*K*) <*c*(*K*\{*k*}) + *c*({*k*})

This is very useful as the set *V' *is simply the set for which *c*_*ik *_is positive.

We also notice that it is possible to divide the problem by using the following lemma

**Lemma 2**: If *J *{1..*N*} and *K *⊂ {1..*N*} (*J *∩ *K *= ∅) are such that ∀_*j*∈*J*_∀_*k*∈*K*_*c*_*jk *_< 0, then there does not exist ∅ ⊂ *J' *⊆ *J *and ∅ ⊂ *K' *⊆ *K *such that *J' *∪ *K' *∈ I*.

**Proof**: Follows from *c*(*J' *∪ *K'*) <*c*(*J'*) + *c*(*K'*)

This means that only connected components are optimal so we only consider connected sets when generating *I *(where we define *i *and *j *to be connected if *c*_*ij *_≥ 0). Also when a term has been removed from the search space (i.e. is no longer in *J *∪ *V*), this may result in previously connected components becoming disconnected. For this reason we also search for the connected components every time we remove an element from the search space. Table 1 shows the number of synsets generated by by using the branch and bound heuristic (lemma 1), the connected components heuristic (lemma 2), finding the connected components then using branch and bound and the final algorithm as follows (shift(*X*) returns and removes the first element of *X*):

**Table 1:** Number of synsets generated by different heuristics

No Heuristics	4.0 × 10^90^
Branch & Bound	5,102,802
Connected Components	1,909,264
CC then B&B	16,629
Final Algorithm	1,689

### Matrix Generation Algorithm

1. *J *= ∅

2. For each connected component *V *in {1... *N*}

(a) Sort *V *by ∑_*k *= 1...*N*_*c*_*ik*_

(b) generate_matrix(*J*, *V*)

function **generate_matrix**(*J*, *V*)

1. While length(*V*) > 0

(a) *k *= shift(*V*)

(b) if(|J| = 0 or ∑_*i*∈*J*_*c*_*ik *_> 0 or -∑_*i*∈*J*_*c*_*ik *_<*max*_*V'*⊂*V *_∑_*i*∈*V'*_, *c*_*ik*_)

i. add *J *∪ {*k*} to *I*

ii. generate_matrix(*J *∪ {*k*}, *V*)

(c) if(*J *∪ *V *has more than one connected component)

i. for each connected component, *C*, with |*C *∩ *J*| > 0 and |*C *∩ *V*| > 0: generate_matrix(*J*, *C *∩ *V*)

ii. end

### Solving the synset problem

This problem is in fact the set covering problem, which is NP-complete, however we found exact methods to be suffcient for our problem. To solve this problem, after we have generated *I *= {*I*_1_,... *I*_*n*_} we formed a matrix *A *= (*a*_*ij*_), given by

$$a_{ij}=\{\begin{array}{ll} 1 & \text{if }j\in I_{i};\\ 0 & \text{otherwise} \end{array}$$

We also form a vector *c *where *c*_*k *_= *c*(*I*_*k*_) and now finding an exact cover is equivalent to finding the integer vector, *x*, which maximises *c*^*T*^*x *subject to

*Ax *= 1

This is a well studied problem, and finding this vector *x *can be viewed as a branch and bound problem and attacked through the Dancing Links algorithm [18]. This algorithm uses a sparse matrix formation to efficiently remove infeasible row choices, and combined with a branch and bound algorithm can very effectively find optimal solutions. The branch and bound simply discards any partial solution when the most it costs is guaranteed to be less than the best solution found so far. This maximum cost can be found by simply tabulating the best clique available for each unsatisfied column in the matrix. More advanced algorithm use either linear relaxation (that is allowing *x *to take non-integer values and solving with the simplex algorithm), or *Lagrangian relaxation with sub-gradient optimisation *[19] to estimate this upper bound, however we did not find this necessary for our data sets.

Regardless of the implementation of the solver one general purpose improvement is to attack each of the sub-problems (i.e. each of the connected components) separately, and then combine the results. The number of ways of partitioning a set is given by Bell's number

$$B_{n}=\sum_{k=0}^{n-1}\left(\begin{array}{l} n\\ k \end{array}\right)B_{k}$$

Bell's number gives the worst case search space size for the dancing links algorithm. If we can divide the problem into connected components, given by *S *= {*S*_1_,... *S*_*n*_} then the size of the search space is

$$\prod_{S_{i}\in S}B_{\left|S_{i}\right|}$$

However by attacking each of the problems separately we get the size of the search space as

$$\sum_{S_{i}\in S}B_{\left|S_{i}\right|}$$

Therefore it now follows that the complexity of this problem is primarily dependent on the size of the largest connected component. In a standard Erdős-Rényi random graph this grows logarithmically if the probability of an edge existing between a pair of nodes is less than *n*^-1 ^as in our problem. Although our problem is not truly equivalent to an Erdős-Rényi random graph, the false positive links between different synsets can be reasonably modelled by an Erdős-Rényi graph. Still the complexity of the largest connected component *B*_*c *log(*n*) _is not polynomial so it is still possible that we may not be able to find the optimal solution for larger test sets, however this would only affect the largest connected components, which as the test set grows larger would represent fewer of the terms and so fewer of the links. In this case we can solve most of the problem exactly, and some of it approximately to give a good overall solution.

## Results

### Comparison to Encyclopedia/Thesaurus

To provide a comparison of our results we used encyclopedia and thesauri. We chose to use 4 sources: these sources were Wikipedia following the method suggested in [20], WordNet [21] the widely used machine readable thesaurus and two domain specific resource Medline Encyclopedia [22] which contains a specialised thesaurus on the subject of human diseases, MeSH [23], a very large controlled vocabulary containing synonyms for a huge variety of medical terminology and UMLS [24], a meta-thesaurus. MeSH, UMLS and WordNet were queried using their built in tools so differences in spelling, pluralisation were considered. As Wikipedia and Medline encyclopedia were not originally intended to be used as thesauri we had to adapt them as follow

1. **Wikipedia (redirect)**: Wikipedia uses a redirect table for terms which should direct to the same article, if this is used the text "(Redirected from ...)" appears beneath the title. Hence we classified two terms as synonyms if they redirected to the same page. For example (example works as of 7 November 2007) the Wikipedia page for "WNV" is the same as the page for "West Nile virus"

2. **Wikipedia (search)**: An alternative way to use Wikipedia is to take the top search term using Wikipedia's search engine. Again we classified two terms as the same if they had the same top search result.

3. **Medline Encyclopedia: **Many articles in the Medline Encyclopedia, had an "Alternative Names" section, which combined with the title of the article gave us synsets. This gave us a good set of medical terminology however it did not cover animals and animal diseases.

All of our test synonym sets, which we found from the 150 abstracts containing the term "infectious diseases", were queried against these resources and the number of links correctly found are presented in Table 2. We found that these encyclopedia and thesauri made a number of mistakes

**Table 2:** Encyclopedia Results (with standard error at 90%)

	Precision	Recall	F-Measure	Coverage
Wikipedia (redirect)	46.4 ± 10.9%	18.8 ± 5.7%	26.8 ± 7.1%	54.1 ± 4.7%
Wikipedia (search)	40.1 ± 8.5%	24.6 ± 6.3%	30.9 ± 6.8%	56.1 ± 4.7%
WordNet	100 ± 0.0%	6.9 ± 3.7%	13.0 ± 6.4%	38.0 ± 4.6%
Medline Encyc.	66.7 ± 30.4%	4.0 ± 2.9%	7.5 ± 5.2%	28.1 ± 4.3%
MeSH	61.6 ± 13.8%	15.8 ± 5.3%	25.2 ± 7.4%	55.1 ± 4.7%
UMLS	94.0 ± 4.8%	46.5 ± 7.3%	62.3 ± 6.8%	79.7 ± 3.8%

1. Failing to find a term

2. Producing an entry for the disease caused by the virus, bacteria etc. or *vica versa*

3. Producing a match that was totally irrelevant e.g. FPV, an abbreviation of "feline parvovirus" in bio-medicine, but Wikipedia instead produces a page on "Ford Performance Vehicles" (as of 24 July 2007, after our data was obtained, a disambiguation page is shown).

4. Matching a term that was broader or narrower than the one requested e.g. vCJD was matched as CJD

5. Some disagreement about synonymy of terms, see the guidelines at the beginning of the data collection section.

### Experiment Results

First for our evaluation we used a naïve classifier we will call the *occurrence *classifier, which simply decided that two terms were synonymous if they co-occurred in any of our patterns. This unsurprisingly gives a very low precision but also the recall is only 63.4%, which will limit the recall of any method we try to use on this data. We found that 78.3% of these non-occurring term pairs, involved one or more term which matched less than 100 articles on PubMed. This suggests that this value may be very close to a limit of the recall of the method.

**Figure 3:**
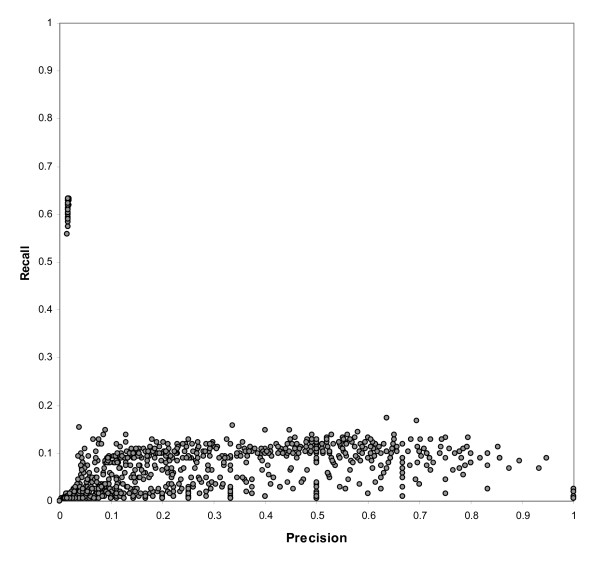
**Precision/Recall by Pattern**. A scatter chart of precision vs recall for all generated patterns. Each point on this corresponds to a pattern generated from the test set and its recall and precision when used as the only variable for a classifier.

Table 3 lists the performance of some single patterns and gives us a baseline for our method and Figure 3 shows the spread of recall and precision for all generated patterns. It can be seen that most of these are variation on parentheses apposition patterns also suggested in Yu *et al *[12]. We also listed a number of patterns that were domain-specific, to show the value of generating patterns for each specific domain. Finally we examined three more patterns from Yu *et al *which did not perform so well in our experiments, this was partly due to our syntax-free approach matching sub-terms.

**Table 3:** Individual pattern results and example matches (*†*: suggested in Yu *et al *[12]; *erroneous term pair; *terms identified*)

Pattern	Precision	Recall	F-Measure
* of # (* * * #) * *	63.6 ± 10.5%	17.3 ± 4.4%	27.2 ± 6.0%
	overview of *avian influenza *(a.k.a. *bird flu*) and phases		
# (#)*†*	79.4 ± 11.4%	13.4 ± 3.9%	22.9 ± 6.0%
	*community-acquired MRSA *(*CA-MRSA*)		
of * # (* #) * *	65.1 ± 11.8%	13.9 ± 4.0%	22.9 ± 6.0%
	of non-*severe acute respiratory syndrome *(Non-*SARS*)-related human		
and # * (# *	75.0 ± 11.8%	13.4 ± 4.0%	22.7 ± 6.0%
	and *hepatitis C virus *infection (*HCV*) and		
*, * # * (# * *	60.0 ± 11.9%	13.4 ± 4.0%	21.9 ± 5.8%
	septicemia, anthrax, *swine *fever (*hog *cholera), and		
against # (* #	64.0 ± 15.9%	7.9 ± 3.1%	14.1 ± 5.2%
	against *enterotoxigenic Escherichia coli *(or *ETEC*		
prevalence of # * #	26.9 ± 8.8%	8.9 ± 3.3%	13.4 ± 4.7%
	*prevalence of *FeLV *and *FIV *prevalence of *paratuberculosis *(ie, *Johne's Disease*)		
patients with # * * * * # * *	12.8 ± 5.0%	7.4 ± 3.0%	9.4 ± 3.7%
	patients with *HTLV-associated myelopathy *(HAM)/tropical spastic paraparesis (*TSP*)		
* * # * known as #*†*	20.0 ± 33.8%	0.5 ± 0.8%	1.0 ± 1.6%
	*an atypical *pneumonia*, also known as *severe acute respiratory syndrome*		
#/#*†*	25.0 ± 21.4%	1.5 ± 1.4%	2.8 ± 2.6%
	*HIV/*AIDS HTLV-associated myelopathy*/*tropical spastic paraparesis*		
#, #*†*	1.7 ± 0.2%	63.4 ± 5.6%	3.3 ± 0.3%
	*measles,*mumps*, rubella vaccine due to efforts to eradicate *poliomyelitis*, *polio *cases have fallen		

We then tried several statistical pattern recognition algorithms (we used the WEKA implementations [25] in all cases), finally grouping the results into synsets based on the outputted probability of the logistic regression and SVM regression classifier by the method described above. The pattern generation took 51 hours on an Dual Core 1.66 GHz processor with 512 MB of memory, then a further 33 hours to generate feature vectors, but we feel that this could be vastly improved by the use of some kind of indexing of the corpus. The classification took 85 seconds and the synset formation about 5 seconds when based on the SVM Regression data and about 127 seconds when based on the logistic regression probabilities, this was due to more positive links in this data set, hence a larger maximum connected component.

**Figure 4:**
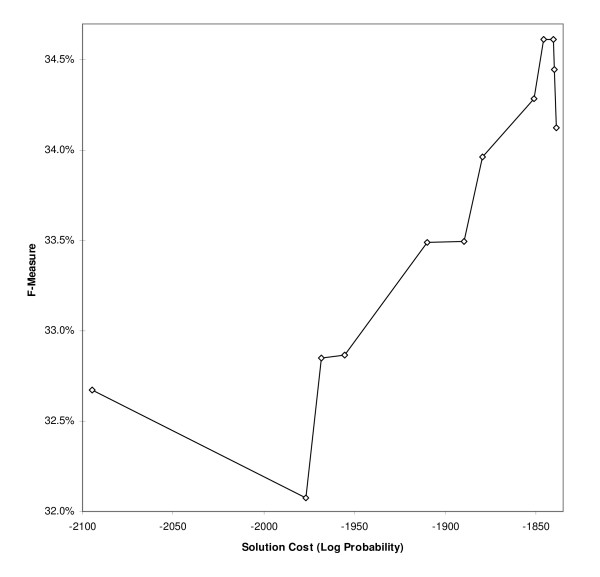
**Solution cost versus F-Measure**. Chart illustrating increase of F-Measure versus solution cost. The data points are the synset solutions generated by the synset solver before finding the optimal solution and the optimal solution. The chart shows their cost and true F-Measure against the test set.

In Figure 4 we calculated the experimental results that would be outputted if we stopped the synonym set solver before we had found the optimal solution. This illustrates that the theoretical cost *c*(...) is correlated to the experimental F-Measure, so better theoretical solutions produce better actual results. Looking at the main results (Table 4) we see the grouping the results in synsets improves the results in terms of totally synonymous results, and although the standard error is large we find the difference is significant at a 99% level using the p-test as described in Yeh [26]. Also the results after synset grouping appeared to be closer, so we analysed the results according to the degree of relation

• Modified: Terms differed by inclusion of a modifier

• Variant: We defined this precisely as any organism term referring to organisms of the *same species*, for example "feline infectious peritonitis virus" which is a mutation of "feline enteric coronavirus". Also we defined this as diseases caused by the same agent but at different stages of progression, for example "dengue fever", which may develop into "dengue hemorrhagic fever"

• Method of Transmission/Point of Infection (MoT/PoI): These are diseases (and agents causing them) which differ only in method of transmission, for example "hospital-acquired", or point of infection.

• Agent/Disease: One term refers to a disease and the second to an agent causing that disease

• Hypernym: The terms showed a clear hypernym/hyponym relation that was not covered by the above groups.

**Table 4:** Results by classifiers (with standard error at 90%)

	Precision	Recall	F-Measure
Occurrence	1.7 ± 0.2%	63.4 ± 7.0%	3.3 ± 3.7%
Naïve Bayes	30.6 ± 5.3%	37.5 ± 7.1%	33.8 ± 5.7%
Logistic Regression	40.8 ± 7.6%	29.7 ± 6.6%	34.2 ± 6.6%
C4.5	74.1 ± 11.7%	21.3 ± 6.0%	33.1 ± 7.8%
SVM	82.2 ± 10.5%	22.8 ± 6.1%	35.7 ± 7.9%
Logistic Regression & Synset Formation	39.6 ± 6.7%	35.6 ± 6.9%	37.5 ± 6.3%
SVM & Synset Formation	73.2 ± 9.8%	29.7 ± 6.7%	42.3 ± 7.7%

We then analysed the logistic regression result (see Table 5) as the high precision of the SVM based result made this analysis less informative. We can clearly see that although both methods had similar precisions, the term pairs produced by the binary classification were more likely to be unrelated. This is not surprising as erroneous links between terms are likely to be caused by artifacts in the data and method and so be nearly totally at random. In contrast we would expect the pattern-based classification system to be more likely to mistake close synonyms for true synonyms, and so these results should be more consistent in relation to our definition of synset formation. So the reduction in error and an increase in number of near synonyms provides evidence for the validity of our model.

**Table 5:** Analysis of Errors

	Correct	Modified	Variant
Binary	40.8%	1.4%	7.5%
Synset	39.6%	0%	12.1%

MoT/PoI	Agent/Disease	Hypernym	Error

7.5%	12.9%	2.0%	29.3%
9.9%	17.6%	3.3%	17.6%

Table 6 illustrates some sample output from the process.

**Table 6:** Examples of automatically generated synsets

["CA-MRSA", "community-acquired MRSA", "methicillin-resistant Staphylococcus aureus", "MRSA", "methicillin-resistant Staphylococcus" ] – *MoT/PoI errors*
["Litopenaeus vannamei", "shrimp" ] – *Hypernym relations*
["Yersinia pestis", "Plague" ] – *Disease/Agent errors*
["dengue shock syndrome", "DHF", "dengue hemorrhagic fever", "dengue", "dengue fever" ] – *Variant terms*
["rubella", "mumps", "measles" ] – *Other relation (there is a widely-used triple vaccine for these diseases)*

## Discussion

In this work we decided to use terms that we found from actual texts as opposed to using a taxonomic thesaurus such as WordNet. This meant that it was difficult to create a test set and as we only found 101 synonymous term pairs we ended up with very large standard errors. It should be noted that for results with low recall the number of results found was so small that we would require a very large test set to accurately estimate the precision. However we found that our results are in line with previous work on both manual and automatic pattern discovery and this indicates that a good result is still obtainable without the use of syntactic knowledge or very large test sets.

Our pattern generation method developed a number of interesting patterns and identified parentheses as the strongest indicator of synonymy (which by itself scored 22.9% F-Measure, see Table 2). It also found several domain specific patterns suggesting the effectiveness of generating separate patterns for specific domains. Syntax-free patterns were also generated allowing application to resource-poor languages, however this does rely on the terms which we wish to find having already been identified. In our experimental setup we inputted terminology that had been manually extracted from PubMed abstracts. There exist many named entity extractors which would allow us to completely automate the whole process and develop a large set of noun phrases for our method to classify, hence we could automatically develop a large thesaurus which would be easy to manually check for errors. One of the more surprising results we saw was that most of the patterns we generated seemed to be of quite high precision, which was unexpected as the scoring metric we chose to use in our search was biased towards patterns with a high recall. The question of how we could generate patterns with higher recall is an interesting one and deserves further study.

## Conclusion

We conclude that for domains with a large amount of specific vocabulary most of the resources we studied perform worse than the automatic method we have developed here. Also given the amount of effort required to manually construct a resource, automatic thesaurus construction may prove more useful in many situations, either to aid construction or in replacement of manual construction. More importantly we have shown that we can easily automatically find patterns and we do not require any prior knowledge of the language's grammar in order to do this. Even though the patterns we generated were weak by themselves we showed that by statistically combining them we can get a much stronger result. We have also shown that we do not need to know a large number of synsets to develop an accurate classifier; this implies most importantly that this method can be used quickly on a different language. We tested our method on only a limited domain but we feel it would likely generalize well to other domains. Our novel synset grouping method not only converted the result to something more applicable, but also improved on the results for both a strict definition of synonymy, and a more relaxed definition.

We think given the logical/probabilistic framework for synset formation that the model we described should be applicable to other relations (for example hypernymy) and even combinations of relations. However solving this model efficiently and whether this will improve the result for actual data is an issue for future work.

## Authors' contributions

JM participated in the design and conception and carried out the data collection, implementation, analysis of data and drafted the manuscript. NC participated in the design and conception and helped to draft the manuscript. Both authors read and approved the final manuscript
